# Nonmydriatic Photographic Screening for Diabetic Retinopathy in Pregnant Patients with Pre-Existing Diabetes in a Safety Net Population: 1 Year Results from the Diabetic Retinopathy in Pregnant Patients Study

**DOI:** 10.1089/whr.2020.0082

**Published:** 2020-09-28

**Authors:** Malini Veerappan Pasricha, Jodi So, David Myung, Andrea Jelks, Carolyn K. Pan

**Affiliations:** ^1^ Byers Eye Institute, Stanford University School of Medicine, Palo Alto, California, USA.; ^2^ Department of Ophthalmology and Santa Clara Valley Medical Center, San Jose, California, USA.; ^3^ Department of Obstetrics and Gynecology, Santa Clara Valley Medical Center, San Jose, California, USA.

**Keywords:** diabetic retinopathy, health care economics, retinal screening, safety net hospital, telemedicine, vision

## Abstract

***Background:*** Pregnant patients with pre-existing diabetes mellitus (DM) are at increased risk for development or progression of existing diabetic retinopathy (DR). A quality improvement project was initiated to improve DR screening during pregnancy at a safety net hospital. This article highlights the utility and generalizability of our telemedicine-based screening model.

***Materials and Methods:*** In April 2018, we implemented a photographic retinal screening system in the Maternal Fetal Medicine (MFM) clinic at Santa Clara Valley Medical Center in San Jose, CA. The system is intended to screen all pregnant patients with pre-existing diabetes (type 1 and 2). Retinal images are automatically uploaded to a secure server and interpreted by a retina specialist (C.K.P.).

***Results:*** A total of 71 pregnant patients with pre-existing DM were seen in the MFM clinic during the study period. Sixty-six of 71 patients (93.0%) were screened compared with 69.1% in the year prior. Of the 64 patients screened with readable images 11 (17.2%) had DR, whereas 53 did not. Forty-nine of the 64 (74.2%) patients screened underwent screening using the new nonmydriatic system in the MFM clinic. Only 7 out of 47 (14.9%) patients with readable images in the MFM clinic required referral to the ophthalmology clinic.

***Conclusion:*** Our model for DR screening in pregnant patients in safety net hospitals is effective in improving screening rates and expediting evaluation and treatment for those in need. This system can prevent irreversible vision loss in pregnant patients and provides an effective framework for ophthalmic care in a safety net hospital system.

## Introduction

Early regular eye screenings are important for pregnant patients with pre-existing type 1 or type 2 diabetes mellitus (DM), as pregnancy significantly increases the risk for rapid development and progression of diabetic retinopathy (DR).^1–3^ DR is one of the most common and severe ocular sequelae of DM, and remains the primary cause of vision loss and blindness in the United States among adults aged 18–64 years, including women of childbearing age.^4^ Although the exact pathophysiological mechanism of new-onset or progressive DR in pregnancy is unknown, hormonal and hemodynamic changes to the retinal microenvironment and vasculature may play a role.^3,5,6^ Risk factors for worsening DR during pregnancy include duration of diabetes, lack of treatment and poor glycemic control before conception, pre-existing retinopathy, and hypertension.^3^ Strategies to manage and treat pregnancy-related DR focus primarily on early identification and management to prevent irreversible vision loss.^7^ To reduce the risk of progressive DR and vision loss during pregnancy, the American Academy of Ophthalmology (AAO) and American Diabetes Association (ADA) recommend counseling for all diabetic patients seeking pregnancy on their increased risk of DR, and at least an examination during the first trimester.^8,9^ Patients with no retinopathy or mild to moderate nonproliferative DR (NPDR) should receive additional follow-up every 3–12 months, and those with severe NPDR or proliferative DR (PDR) should receive follow-up every 1–3 months.^8^

In states such as California, where low-income pregnant patients automatically qualify for insurance coverage through Medicaid, pregnancy offers a unique window for health interventions as these patients have significantly increased access to care and care utilization during their pregnancy.^10^ Many women may receive their first hemoglobin A1c test and subsequent diagnosis of DM during their first prenatal visit, allowing for disease intervention both during and after their pregnancy. In addition, pregnant patients are also more motivated, engaged, and active in their own health care compared with the general population.^11–13^ Thus, incorporating early regular DR screenings into comprehensive prenatal care is especially important for pregnant patients at safety net hospitals.

Designing and implementing a DR screening protocol specifically for pregnant patients at safety net hospitals leverages these unique patient characteristics and circumstances. Nonmydriatic retinal photography, although not a substitute for a comprehensive eye examination by an ophthalmologist, is an effective and convenient method to screen for retinal disease, including DR.^14^ Studies in multiple care settings have shown that screening protocols utilizing nonmydriatic retinal photography with remote image reading *via* telemedicine can reliably screen for DR.^15–19^ They simultaneously increase screening rates, improve clinical outcomes, and increase overall patient satisfaction. This is mainly because this method reduces barriers to care, including cost, transportation, time constraints, and lack of access to ophthalmologists in the community, as it can be performed by primary care providers during a routine checkup.^20–29^ Prior efforts to improve DR screening among specific populations of pregnant patients have effectively increased screening rates^30,31^; however, these studies do not provide quality improvement guidelines for clinics seeking to iterate and improve their screening strategies over time, and lack generalizability to under-resourced settings such as safety net hospitals.

The Diabetic Retinopathy in Pregnant Patients (DRIPP) study is a quality improvement project aimed at improving DR screening in pregnant patients with pre-existing diabetes in safety net hospital health care systems. At the Santa Clara Valley Medical Center (SCVMC, a safety net hospital), all pregnant patients with pre-existing diabetes in the hospital system (main hospital and affiliated satellite health centers) are referred to the Maternal Fetal Medicine (MFM) clinic for their diabetes and prenatal care. Before this quality improvement initiative, pregnant women with diabetes were referred to the endocrine clinic, where they underwent digital photography retinal screening, or directly to the ophthalmology clinic. However, patients often waited 6–12 months before being evaluated due to the high volume of referrals, and thus did not receive screening within the recommended time frame or proper management of disease. Using evidence-based intervention protocols, a nonmydriatic photographic system was implemented to evaluate pregnant patients with pre-existing diabetes and identify individuals requiring expedited referral to ophthalmology. We aim to evaluate the utility of this project, focusing on potential improvements in DR screening rates and changes in specialty clinic referral patterns, and report our 1 year results.

## Materials and Methods

In April 2018, we implemented a nonmydriatic photographic retinal imaging system (CenterVue^®^ DRS camera) in the MFM clinic at SCVMC. A brief training session was conducted for MFM clinic staff (licensed vocational nurses or medical assistants) who were able to operate the automated retinal camera independent of ophthalmology-trained personnel. Each patient is to remain in a darkened room for ∼1–2 minutes before the image is attempted, to allow the pupils to dilate naturally. No pharmacological dilation is used. The nonmydriatic operation requires a minimum pupil size of 3.8 mm. The patient is instructed to place her chin on the motorized chinrest, which facilitates automatic alignment. She is then requested to fixate on the camera lens and minimize movement to the best of her ability. The room is to remain as dark as possible (*e.g.*, room lights off, natural light obstructed) during image capture. The machine captures two retinal photographs for each patient (one image of each eye) at a resolution of 48 pixels/degree and a single field of 45° × 40°. The camera operates largely independent of an operator with a motorized ability to sequentially move from image capture of one eye to the other, center on a point halfway between the temporal edge of the optic disk and the fovea for each image, and autoadjust the focus and light exposure.

The protocol for DR screening is intended to minimize disruption of workflow in the MFM clinic and allow screening during routinely scheduled prenatal visits. An electronic order for in-clinic nonmydriatic fundus photography is placed by the MFM provider (physician, physician assistant, or nurse practitioner) for every new diabetic patient seen in the MFM clinic. During the same visit or a subsequent visit, the patient is directed to the nonmydriatic photography system to complete the DR screening. Completed photographs are automatically uploaded to a secure server and read by a board-certified retina specialist (C.K.P.) in the SCVMC ophthalmology clinic. Image grading is based on the Airlie House classification and the ETDRS Manual of Procedure.^32^ The retina specialist formulates a follow-up plan based on grading of the retinal photos. Those with severe NPDR, PDR, questionable diagnoses, or features concerning for other diseases unrelated to diabetes are marked for referral to the ophthalmology clinic for further consultation and treatment if appropriate by an eye care specialist (optometrist or ophthalmologist). Others are recommended for repeat photography at the time interval dictated by the AAO screening guidelines.^8^ Recommendations for follow-up or referral are documented in the shared electronic medical record. Any patients requiring referral are scheduled in the ophthalmology clinic.

The Institutional Review Board (IRB)/Ethics Committee deemed this a quality improvement project and all related chart review exempt from IRB approval. Since all pregnant patients seen in the MFM clinic undergo obstetric ultrasounds as part of their early prenatal care, we queried the ultrasound database for patients with a diagnosis of diabetes seen in the MFM clinic during the 1-year period of April 16, 2018 through April 15, 2019. Eligible patients included pregnant patients with a diagnosis of type 1 or type 2 DM of any disease duration before conception. Patients with gestational diabetes were excluded, as this category of patients are not included in the AAO's official guidelines for DR screening.^8^ Our primary outcomes were rate of DR screening and mode of assessment for those who underwent screening, in the study year (April 2018 to April 2019) and in the year prior (April 2017 to 2018). We also reviewed electronic medical records to collect data on patient age, race, initial hemoglobin A1C (earliest available in pregnancy or just before conception), and gestational ages at first prenatal visit and at screening visit. Initial hemoglobin A1C was determined to be an effective proxy for a patient's risk of DR, as opposed to hemoglobin A1C at the time of screening, because patients undergo an intensive glucose control program with the MFM clinic once their DM status and diagnosis are established at their first visit. Finally, we collected data on the prevalence of DR in the study population, follow-up plan patterns, and clinical course of those referred to the SCVMC ophthalmology clinic.

## Results

A total of 71 pregnant patients with pre-existing DM (15.5% Type 1 and 84.5% Type 2) were seen in the MFM clinic during the study period. The average age was 31.8 years, and the population was predominantly Hispanic (70.4%). The average periconception hemoglobin A1C was 8.2% (Table 1).

**Table 1.** Baseline Data for Diabetic Patients Seen in Maternal Fetal Medicine Clinic in First Year of Nonmydriatic Camera Implementation (*n* = 71)

Age (years), mean	31.8
Race	Hispanic = 50/71 (70.4%)
	Asian = 7/71 (9.9%)
	White = 6/71 (8.5%)
	Black = 4/71 (5.6%)
	Pacific Islander = 2/71 (2.8%)
	Dominican = 1/71 (1.4%)
	Native American = 1/71 (1.4%)
Diabetes type	Type 1 = 11/71 (15.5%)
	Type 2 = 60/71 (84.5%)
Hemoglobin A1C (%),^a^ mean	8.2% (Range: 4.8–12.9)
Gestational age (weeks),^b^ median	8.4 (Range: 4.1–21.3)

^a^ At initial MFM visit (or most recent prior).

^b^ At initial MFM visit.

DR, diabetic retinopathy; MFM, Maternal Fetal Medicine.

The rate of overall screening for DR was 66/71 (93.0%). Average gestational age at the time of screening was 13.4 weeks, with the majority undergoing screening during the first and second trimester (40.9% in first trimester, 50.0% in second trimester, and 9.1% in third trimester). Among those screened (*n* = 66), 49 (74.2%) underwent nonmydriatic photography with the newly implemented system in the MFM clinic, 13 (19.7%) underwent in-person examinations in the optometry or ophthalmology clinics, and 4 (6.1%) had pre-existing arrangements for nonmydriatic photography in the endocrine clinic (Table 2). Among the five (7.0%) patients who were not screened, two had an active order that was not completed, and three declined screening in the MFM clinic or did not show to scheduled ophthalmology appointments.

**Table 2.** Diabetic Retinopathy Screening Results for Patients Seen in Maternal Fetal Medicine Clinic in First Year of Nonmydriatic Camera Implementation (*n* = 71)

	Study year (April 2018–2019)	Year prior (April 2017–2018)
Rate of overall screening	66/71 (93.0%)	47/68 (69.1%)
Screened by nonmydriatic camera in MFM Clinic	49/66 (74.2%)	—
Screened by ophthalmology clinic	13/66 (19.7%)	22/47 (46.8%)
Screened by nonmydriatic camera in endocrine clinic	4/66 (6.1%)	25/47 (53.2%)
DR status (*n* = 64^a^)		
No DR	53/64 (82.8%)	38/45 (84.4%)
DR present	11/64 (17.2%)	7/45 (15.6%)
Follow-up plan patterns after nonmydriatic screen (*n* = 47^a^)		
Repeat photograph (or routine eye clinic follow-up) in third trimester or postpartum	40/47 (85.1%)	—^b^
New referral to ophthalmology clinic	7/47 (14.9%)	—^b^

^a^ Two patients screened had unreadable images and no repeat photos were completed to determine pathology and follow-up plan.

^b^ Follow-up protocol did not follow patterns established by the MFM screening initiative. All 22 patients screened in the ophthalmology clinic would continue follow-up there, whereas 4 of 25 patients screened in the endocrine clinic received new referrals to ophthalmology.

In contrast, overall DR screening rate in the year prior (April 2017 to April 2018) to implementation of the nonmydriatic photography system in the MFM clinic was 47 out of 68 total pregnant diabetic patients seen (69.1%). Among the 47 patients screened, 25 were screened in the endocrine clinic using a nonmydriatic photography system housed there, whereas 22 were examined in the ophthalmology clinic (Table 2).

Of the 66 patients screened in the first year after implementing the nonmydriatic camera in the MFM clinic, 11 had some grade of DR: 3 were diagnosed using the MFM nonmydriatic system, and 8 were diagnosed on examination in the optometry or ophthalmology clinic (4 were already being followed before pregnancy for diabetes-related eye complications, 2 underwent initial screen with optometry at a satellite clinic, and 2 were referred to ophthalmology due to unreadable images on 2 attempts in the MFM clinic). Fifty-three of the patients screened had no DR: 37 using the new MFM nonmydriatic system, 12 were screened in the optometry or ophthalmology clinic, and 4 by the nonmydriatic system in the endocrine clinic (Table 2). Two patients screened had unreadable images without subsequent photos to determine pathology.

To assess the quality of the MFM nonmydriatic system, we also analyzed the number of unreadable images produced over the 1 year study period. Of the 49 patients screened using the MFM nonmydriatic system, 10 (20.4%) had unreadable images in one or both eyes. Of these patients, four had readable images on second attempt and two did not undergo repeat photography. Four had unreadable images on two attempts—one was able to produce readable images on a third attempt, whereas the other three were newly referred to the SCVMC ophthalmology clinic.

Follow-up plans for patients screened using the new MFM nonmydriatic system was determined at the time of photograph interpretation by the retinal specialist and conveyed to the MFM provider *via* electronic medical record. Among the 47 patients screened with readable images using the MFM nonmydriatic system, 40 patients (85.1%) had no or mild-moderate DR, and were advised to repeat photographic screening in the MFM clinic in the third trimester of pregnancy or postpartum (or continue routine care in the eye clinic if already an established patient). Seven patients (14.9%) were newly referred to the SCVMC ophthalmology clinic for further evaluation of DR (*n* = 3), findings unrelated to DR (*n* = 2), or persistently unreadable images (*n* = 2). The average number of days between referral from the MFM clinic and appointment in the ophthalmology clinic was 40.

Among the 11 patients who screened positive for DR, 3 (4.7%) developed significant progression during pregnancy necessitating treatment with laser and/or steroid eye injections. Two of these patients were already being followed in the ophthalmology clinic and one was referred after screening in the MFM clinic. Two other patients demonstrated progression of existing DR, but did not require treatment. It is unknown whether the remaining six patients with DR were manifesting new or progression of existing DR, as they did not have preconception eye examination data.

## Discussion

Our findings demonstrate the successful implementation of a nonmydriatic retinal screening protocol for pregnant patients with pre-existing diabetes at a safety net hospital. Sixty-six out of 71 (93.0%) patients were screened, an increase both in rate and magnitude from the year before (47 out of 68 patients, or 69.1%). Of the 64 patients screened with readable images, 11 (17.2%) had some degree of DR, and 53 (82.8%) had no DR.

Nonmydriatic retinal screening offers several important advantages for pregnant patients at a safety net hospital. First, it greatly reduces the appointment burden and increases convenience for patients, as they can receive DR screening during an existing prenatal appointment at the MFM clinic and do not have to undergo dilation. Of the 47 patients screened successfully at the MFM clinic, only 7 (14.9%) required a new referral for follow-up at the ophthalmology clinic, dramatically decreasing appointments and reducing associated financial or time costs for patients such as taking time off work or paying for transportation or childcare.^33,34^

Integrating DR screening into the MFM clinic also greatly reduced the referral burden of other specialty clinics. In the year before placing a nonmydriatic camera at the MFM clinic, 25 out of 47 (53.2%) patients were screened by the endocrine clinic, and 22 (46.8%) by the ophthalmology clinic. After implementing the nonmydriatic camera, 47 out of 64 (73.4%) patients were screened using the nonmydriatic camera in the MFM clinic, 4 (6.3%) by the endocrine clinic, and 13 (20.3%) by the ophthalmology clinic. Of the 13 patients screened by the optometry/ophthalmology clinic, all were either already pursuing care at the ophthalmology clinic for diabetic eye complications or were receiving care primarily at a satellite center that was not equipped with a nonmydriatic screening machine but housed an optometry clinic instead. Similarly, the four patients screened in the endocrine clinic were already undergoing routine care with this specialty. Of note, the utilization of the MFM screening system increased dramatically over the first year of its implementation: only 43.8% of screenings took place at the MFM clinic in the first 3 months of the study, which increased to 85.7% of all screenings by the last 3 months of the first year. In the absence of the MFM screening protocol, all 64 patients would have been seen by specialists at the ophthalmology/optometry clinics or by screening staff at endocrine clinics, demonstrating that integrating DR screening into the MFM clinic approach can reduce the referral burden of pregnant patients with DM to other specialists, and ultimately increase provider capacity and appointment availability for patients who require time-sensitive care. Moreover, integrating first-line DR screening into all MFM appointments opens communication between MFM and ophthalmology providers, and offers an opportunity for providers to improve both care coordination and quality of care.

Image readability also improved over time. Ten (20.4%) of 49 patients screened at the MFM clinic had unreadable images in one or both eyes on first attempt. Although five achieved readable images on repeat attempts and two never underwent repeat photography, three had persistently unreadable images and were among the seven patients who needed new referrals to the ophthalmology clinic. In analyzing trends, the number and percentage of unreadable images rose in the initial 9 months after implementing the new photographic system, then fell in the following 3 months, most likely due to training and increasing experience with the screening protocol of the MFM clinic staff.

Nonetheless, there are several areas for improvement. AAO and ADA guidelines recommend that all patients with pre-existing DM receive screening during a pregnancy.^8,9^ However, five patients (7.0%) never received DR screening: three were due to patient compliance and two did not have initial screening orders placed by the MFM clinic.

Moreover, guidelines recommend that all patients with DM receive screening during their first trimester, but only 27 (40.9%) patients were screened in the recommended time frame.^8,9^ Upon further investigation, we found that screening trimester was influenced by when the patient first presented to the MFM clinic, as most patients who received late screenings established care with the MFM clinic after their first trimester. Fourteen of the 33 patients screened in their second trimester only presented to the MFM clinic in their second trimester; similarly, two out of six of patients screened in their third trimester only presented to the MFM clinic in their second trimester. For many patients under MFM care during their first trimester who did not receive a DR screening until later in their pregnancy, initial prenatal visits were often largely focused on achieving tight control of their diabetes, understanding their pre-existing medical conditions, and addressing socioeconomic barriers, with a particular focus on the health of the pregnancy itself.

Further characterizing the reasons underlying these gaps in screening is needed to develop targeted interventions that can effectively improve screening rates and timing of screenings. Research shows that increasing patient education and awareness is critical for successfully and sustainably increasing DR screening rates.^35–38^ Adopting new strategies to educate patients on the risk of DR in pregnancy may improve patient awareness and increase adherence to screening guidelines. System-wide quality improvement initiatives should also be developed to improve screening rates. Reminders for providers—whether built into the electronic health record, or just on a simple paper checklist—have effectively increased DR screening rates in a variety of clinical contexts, including ours.^35^ Reminders for back office staff to check for an order may also be helpful. Thus, improving screening rates and adherence to guidelines requires a multifaceted approach that comprehensively addresses screening challenges from the perspective of patients, providers, and the overarching health system.

After nonmydriatic imaging, patients requiring follow-up at the eye clinic were scheduled to see an ophthalmologist an average of 40 days after initial referral from the MFM clinic (excluding one patient who was referred to the eye clinic from an external, non-SVCMC facility). Given these patients' time-sensitive needs, we aim to decrease this wait time to <4 weeks by creating a protocol at the ophthalmology clinic to expedite appointments for all patients with DR referrals from the MFM clinic. We anticipate that the long-term decrease in patient referrals resulting from the nonmydriatic screening protocol will increase appointment availability and provider capacity. In addition, several patients who were newly referred to the ophthalmology clinic did not attend scheduled appointments, likely due to limited understanding about the risk of disease progression during pregnancy and the vision-threatening potential of DR. Increasing patient awareness about the importance of screening during early pregnancy through education at the MFM clinic may also improve patient engagement with ophthalmic providers and may motivate patients to attend scheduled eye clinic appointments.

To continue improving image readability, techniques to emphasize are turning off the lights and darkening the room as much as possible before photography, allowing adequate time for the patient's pupils to dilate, and appropriately positioning the patient. Providing a standardized script with patient instructions (*e.g.*, to look at the camera lens and remain still during photography) may also be helpful to standardize the workflow for MFM clinic staff. Unfortunately, several unavoidable factors may decrease image readability, including naturally dilated pupils less than the minimum threshold needed for accurate image capture, poor patient cooperation, and media opacities.^39^ We hope that future advancements in nonmydriatic retinal screening technology will address these factors.

Addressing these existing limitations will increase the generalizability of our DR screening protocol for implementation at safety net hospitals nationwide. DR can be easily and readily identified *via* nonmydriatic retinal photography, but can lead to vision loss and even blindness if it is not discovered and treated early. Moreover, vision loss is associated with significant comorbidities, including decreases in quality of life, independence, mobility, mental health, cognition, social function, and employment.^40^ MFM clinics—in particular, those in under-resourced health care systems, such as safety net hospitals—should consider implementing a nonmydriatic retinal screening program, as it is a highly cost-effective method to both reduce the risk of progressive DR for pregnant patients with diabetes who otherwise may not have access to ophthalmic care, and reduce load and increase provider capacity for patients requiring specialist care. Effective interventions that have successfully increased DR screening rates (through dilated fundus examination) include educating patients and providers on the risks of DR, increasing general population awareness of the risks of DR, and utilizing screening reminders.^35–38,41^ Additional research is necessary to contextualize, design, and evaluate these interventions to improve nonmydriatic retinal screening for pregnant patients, taking into account the specific needs of this patient population.

We are aware that there may be several economic challenges associated with this undertaking. First, there is limited availability of low-cost nonmydriatic imaging systems. Commercially available digital imaging systems and data handling often require a large initial capital investment (minimum $50,000–$100,000). Our camera was donated through our partnership with a private academic institution. Without question, this is not a sustainable means of acquiring such equipment and public funding support will likely be needed for a generalizable program. Alternatively, there are several new cost-effective options for portable nonmydriatic cameras (ranging from $5,000 to $10,000); however, there is still the unknown financial burden related to image and data handling.^42–44^ Second, there is lack of uniform reimbursement policies and most do not provide competitive advantages for the providers involved. Reimbursement from commercial insurance companies, Medicare, and Medicaid for image-based evaluations of DR has been limited in the United States. Finally, there are costs associated with training obstetric staff and using their paid time during clinic hours to adopt screening protocols.^39^

In summary, this study demonstrates that nonmydriatic retinal screening at an MFM clinic can effectively increase DR screening rates, reduce appointment and referral burden, and ultimately improve ophthalmic care for pregnant patients at a safety net hospital. There remain several opportunities for improvement, including further increasing DR screening during the first trimester and identifying barriers to care for patients who are not screened, improving provider workflow, decreasing referral wait times, and improving image readability. Further work is needed to explore the role of multidisciplinary quality improvement interventions such as patient and provider education, provider training, and system-wide reminders for MFM clinic staff to improve DR screening for pregnant patients at safety net hospitals, and will increase the generalizability of the screening protocol for obstetric patients and providers at safety net hospitals nationwide.

## Abbreviations Used

AAO: American Academy of Ophthalmology
ADA: American Diabetes Association
DM: diabetes mellitus
DR: diabetic retinopathy
IRB: Institutional Review Board
MFM: Maternal Fetal Medicine
NPDR: nonproliferative DR
PDR: proliferative DR
SCVMC: Santa Clara Valley Medical Center
